# A competing risk analysis of colorectal cancer recurrence after curative surgery

**DOI:** 10.1186/s12876-022-02161-9

**Published:** 2022-03-03

**Authors:** Angela E. Schellenberg, Veronika Moravan, Francis Christian

**Affiliations:** 1 Department of Surgery, Selkirk Regional Health Centre, 120 Easton Drive, Selkirk, MB R1A 2M2 Canada; 2 Department of General Surgery, University of Saskatchewan, Saskatoon, SK Canada; 3 Applied Statistician, Toronto, ON Canada; 4 Easton Place Clinic, Box 400, 15 Wersch Street, Selkirk, MB R1A 2B3 Canada

**Keywords:** Competing risk, Cumulative incidence function, Colorectal cancer, Recurrence

## Abstract

**Background:**

This study examines the effect of prognostic patient and disease characteristics on colorectal cancer (CRC) recurrence after curative resection. We used competing risk analysis with death as a competing risk. This method provides the clinician a perspective into a patient’s actual risk of experiencing a recurrence.

**Methods:**

A retrospective cohort study of patients diagnosed with CRC who underwent curative resection for CRC from 2003–2007 at the Royal University Hospital in Saskatoon was completed. The outcome of interest was the first CRC recurrence, either local or distant metastasis. Demographic data, tumor characteristics, adjuvant treatment and follow-up data, date of local recurrence or metastasis were recorded from the medical record. Univariate analysis was completed to look at the relationship between each of the prognostic indicators and recurrence. Multivariable modelling (subdistribution regression modelling) was done to identify the main risk factors in determining recurrence.

**Results:**

Of 148 patients, 38 (25.7%) experienced a recurrence, 16 (10.8%) died without evidence of recurrence, and 94 (63.5%) experienced neither outcome. The median follow-up was 30.5 months (interquartile range 10.6–50). In univariable subdistribution regression, T-stage, N-stage, vascular invasion and positive margins were all predictive of cancer recurrence, with *p* ≤ 0.001, with subdistribution hazard ratios for T4 stage at 11.93, T3 stage at 2.46, N2 stage at 10.58, and presence of vascular invasion at 4.27. N-stage remained as the sole predictor in multivariable regression. Cumulative incidence function (CIF) of recurrence at 48 months after surgery was 15%, 27% and 90% for N1/2, N3 and N4 respectively.

**Conclusion:**

The highest CIF of recurrence was associated with T4 stage, N2 stage, and vascular invasion. Patient’s age, tumour location, type, or histological grade were not found to have a significant effect on the success of CRC surgery in precluding a recurrence.

## Background

Every year an estimated 1.4 million people worldwide are diagnosed with colorectal carcinoma (CRC) [1]. In North America, CRC is the second most common cause of cancer-related death that affects both men and women. Canada is among the countries with the highest incidence of CRC [2]. The current primary treatment for CRC is surgical resection with the intent to cure [3]. In a cohort of CRC patients with stage 1—4 disease, locoregional recurrence or metastasis was demonstrated in 26.6% of patients [4]. CRC recurrence was detected in 16.6% of patients with stage 1—3 disease, after a mean of 4.4 years, in a randomized trial comparing various degrees of follow-up [5]. In another study, 30% of CRC patients with stage 1—3 disease developed recurrence following surgery for curative intent [6].

The objectives of our study were to describe the recurrence rates of CRC after surgical resection and to determine which patient level and disease level characteristics were associated with an increased risk of recurrence in follow-up. A CRC recurrence, either local or a distant metastasis, was considered a failure to cure. The traditional Kaplan–Meier survival function results in an overestimate of the absolute likelihood of a recurrence in the presence of deaths prior to recurrence. Therefore, competing risk analysis was used in this study because it provides the clinician a perspective into a patient’s actual risk of experiencing a recurrence. The cumulative incidence function (CIF) was used to estimate the probability of a recurrence over time, treating death as a competing risk. The effects of demographic and disease characteristics as covariates on the CIF was modelled with Fine-Gray subdistribution (SD) regression.

## Methods

This is a retrospective study of patients diagnosed with CRC who subsequently underwent curative resection from 2003–2007. A total of 226 medical charts from the Royal University Hospital in Saskatoon, Canada were reviewed. Patients who received pre-operative chemotherapy or radiation or demonstrated pre-operative evidence of metastatic disease were excluded. Demographics, disease characteristics, surgical approach, and adjuvant treatment were recorded. Pathology reports from the initial surgical resection were reviewed for tumor site, tumor type, histological grade of malignancy, status of margins, TNM staging, presence of vascular and perineural invasion, and degree of lymphocytic reaction. Follow-up data included time to local recurrence or metastasis, time of most recent follow-up visit, and date and cause of death. Patients not followed-up at the hospital were compared to those with follow-up, but excluded from further analysis. This study was approved by the research ethics board of the University of Saskatchewan. Informed consent was not obtained in this retrospective chart review.

Descriptive statistics were tabulated from data collected for 192 patients meeting the inclusion criteria. Characteristics of the 148 patients with follow-up and the 44 patients without were tabulated. The mean (standard deviation) and median (intra-quartile range, IQR) are presented for numeric variables, and frequency (percentage) for categorical variables. Differences between the two groups were tested with t-tests for continuous variables, and Chi-square test, or Fisher’s exact test in presence of cell sizes of 5 or fewer, for categorical variables. Further analysis was restricted to patients for whom follow-up data was available.

The outcome of interest was recurrence of CRC, either local or metastasis, with death prior to recurrence as a competing risk. Censoring time was set as most recent follow-up date. CIF curves of recurrence by each level of prognostic covariates were estimated, charted, and visually inspected. The non-parametric Gray test was employed to test for differences between pairs of CIFs [7].

The effect of covariates on the CIF was modelled using Fine-Gray SD regression [8]. SD regression allows us to estimate the relative effects of patient and disease characteristics as covariates on the risk of recurrence, while taking risk of death into account. Modeling the effect of covariates on the CIF allows one to estimate the effect of covariates on the absolute risk of the outcome over time. Subdistribution hazard ratios (SHR) obtained from the Fine‐Gray model describe the relative effect of covariates on the CIF [9]. Univariable modelling was conducted. The Wald test assessed covariate significance and SHRs with 95% CI were generated. Multivariable modelling was done using forward selection, with Akaike information criterion (AIC) and Bayesian information criterion (BIC) [10]. Plots of Schoenfeld residuals were used to evaluate the proportional hazard subdistribution assumption.

In regression analysis age was centered at 60 and divided by 10, resulting in an increase of one in the SHR for every decade over 60 years of age. Cases with missing values for covariates were excluded from any analysis involving the covariate. All testing used the traditional significance level of α = 0.05. All statistical analysis was done using the R software package, version 4.04 [11]. CIF analysis was done with package *cmprsk2* version 0.0.0.9003 [12], stepwise SD regression with package *crrstep* version 2015–2.1 [13].

## Results

### Case selection

**Fig. 1:**
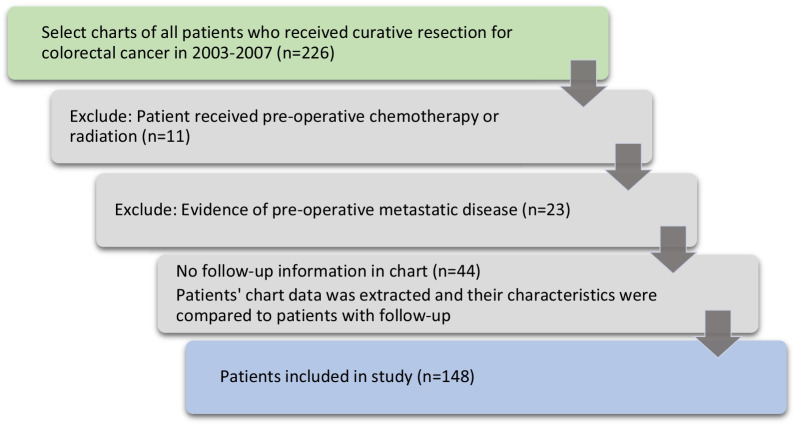
Selection of patient charts for inclusion in study

The selection process for medical charts is shown in Fig. 1. Out of 226 patient charts, 11 were excluded for pre-operative chemotherapy or radiation, and 23 for known metastatic disease prior to surgery. Of the remaining 192 charts, 44 patients lacked follow-up information. Table 1 compares characteristics of patients without follow-up to the 148 patients with follow-up visits. Disease characteristics were similar for both groups; site (*p* = 0.559), tumour type (*p* = 0.125), histology (*p* = 1), T-stage (*p* = 0.5)*,* N-stage (*p* = 0.932), M-stage (*p* = 1), vascular invasion (*p* = *1*), and perineural invasion (*p* = 0.929), as were measures of surgical quality positive margins (*p* = 1) and nodes examined (*p* = 0.319). Patients without follow-up had surgery in the earlier years of the study (*p* = 0.005), and were older 75.7 ± 11.1 years v. 69.2 ± 11.4 years (*p* = 0.001). Post-operative chemotherapy or radiation was recorded for 9.1% v. 31.1% (*p* = 0.003*)*.

**Table 1:** Patient, disease and treatment characteristics for 148 patients in the study and 44 patients excluded due to unavailability of follow-up

Variable	Follow-up available	Follow-up unavailable	Follow-up vs none
	No. (%)	No. (%)	*p* value^1^
All patients	148 (100)	44 (100)	
Age (years) mean ± SD	69.2 ± 11.4	75.7 ± 11.1	0.001
Median (IQR)	71 (62, 78)	78.5 (70, 84.2)	
Male	78 (52.7)	27 (61.4)	0.401
Year of surgery			
2003	27 (18.2)	6 (13.6)	0.005
2004	39 (26.4)	9 (20.5)	
2005	28 (18.9)	18 (40.9)	
2006	27 (18.2)	10 (22.7)	
2007	27 (18.2)	1 (2.3)	
Tumour site			
Colon	59 (39.9)	17 (39.5)	0.559
Rectum	55 (37.2)	13 (30.2)	
Sigmoid/rectosigmoid	34 (23)	13 (30.2)	
Tumour type			
Adenocarcinoma NOS	132 (89.2)	43 (97.7)	0.125
Mucinous adenocarcinoma	15 (1.1)	1 (2.3)	
Carcinoid	1 (.7)	0	
Histological grade			
Low/well differentiated	52 (35.2)	15 (34.1)	1
Moderate	78 (52.7)	24 (54.5)	
High/poorly differentiated	16 (1.8)	5 (11.4)	
T-stage			
T0 (in situ)	0	1 (2.3)	0.5
T1	13 (8.8)	2 (4.5)	
T2	35 (23.8)	11 (25)	
T3	86 (58.5)	26 (59.1)	
T4	13 (8.8)	4 (9.1)	
N-stage			
N0	92 (62.6)	28 (63.6)	0.932
N1	35 (23.8)	11 (25)	
N2	20 (13.6)	5 (11.4)	
M-stage M1	3 (2)	0	1
Vascular invasion	39 (27.3)	12 (27.3)	1
Perineural invasion	40 (27.8)	11 (25.6)	0.929
Positive margins	15 (10.3)	4 (9.1)	1
Nodes examined			
Mean ± SD	15.4 ± 7.4	14.3 ± 5.7	0.319
Median (IQR)	13 (11, 19)	14 (12, 17)	
Post-operative chemotherapy and/or radiation	46 (31.1)	4 (9.1)	0.003

^1^ T-test for continuous variables, Pearson’s χ^2^ test or Fisher’s exact test for categorical variables

These 44 patients were excluded from further analysis.

### Patient and disease characteristics

Table 1 describes the 148 patients in the study. Their mean age was 69.2 ± 11.4 years, with 52.5% being male. The majority of tumours, 89.2%, were adenocarcinoma type. Tumour location was colon 39.9%, rectum 37.2%, sigmoid 19.6% and rectosigmoid 3.4%. Most exhibited low grade 35.2% or moderate 52.7% histology with only 1.8% high grade. Tumour staging was T1 8.8%, T2 23.8%, T3 58.5% and T4 8.8%. Node staging showed N0 62.6%, N1 23.8% and N2 13.6%. Metastasis was discovered in 3 (2%) patients, all cases located in the omentum. Vascular invasion was noted in 27.3% and perineural invasion in 27.8%. Positive margins were realised in 10.3% of patients, and the mean number of nodes examined was 15.4 ± 7.4. Post-operative chemotherapy was given to 17.6% of patients, radiation to 2%, and both to 11.5%.

### Outcomes

Table 2 shows time from curative surgery to the first CRC recurrence, or end of follow-up. CRC recurrence was diagnosed in 38 (25.7%) patients; 7 local, 26 metastasis, and 5 both. Median (IQR) time of recurrence was 19.7 (8.7, 28.2) months. Sixteen (10.8%) patients died with no indication of recurrence. Twelve deaths occurred prior to discharge from hospital, and four under follow-up. Ninety-four patients were disease-free at time of their last follow-up, with median (IQR) follow-up of 44.8 (20.9, 56.6) months.

**Table 2:** Time from curative surgery to first of CRC recurrence, death or end of follow-up

	N	Time to event^1^	
		Median (IQR)	Minimum–maximum
All patients	148	30.5 (10.6, 50)	0–111
Diagnosed with CRC recurrence	38	19.7, (8.7, 28.2)	1.4–87.1
Local recurrence	7		
Local recurrence & metastasis	5		
Metastasis	26		
Died with no evidence of recurrence	16		
Prior to hospital discharge	12	7.5 (3.2, 22.2)	0–39
After discharge from hospital	4	16.7 (11.7, 39.7)	9.4–61.7
Alive with no evidence of recurrence	94	44.8 (20.9, 56.6)	0.4–111

^1^All times shown in months, except for death prior to discharge from curative surgery, shown in days

A total of 12 patients died prior to hospital discharge or shortly after discharge. These patients tended to be older, mean (standard deviation) age 77.9 (9.7) vs 68.5 (11.3), t-test *p* = 0.006, with tumours in the colon, 75% vs 50%, chi-square test *p* = 0.034. Cause of death was documented in four charts: one hypoxia, two from myocardial infarction, and respiratory failure for a patient with chronic obstructive pulmonary disease. Four patients died later without any indication of recurrence. Causes of death were myocardial infarction, stroke, lymphoma, and other causes.

**Fig. 2:**
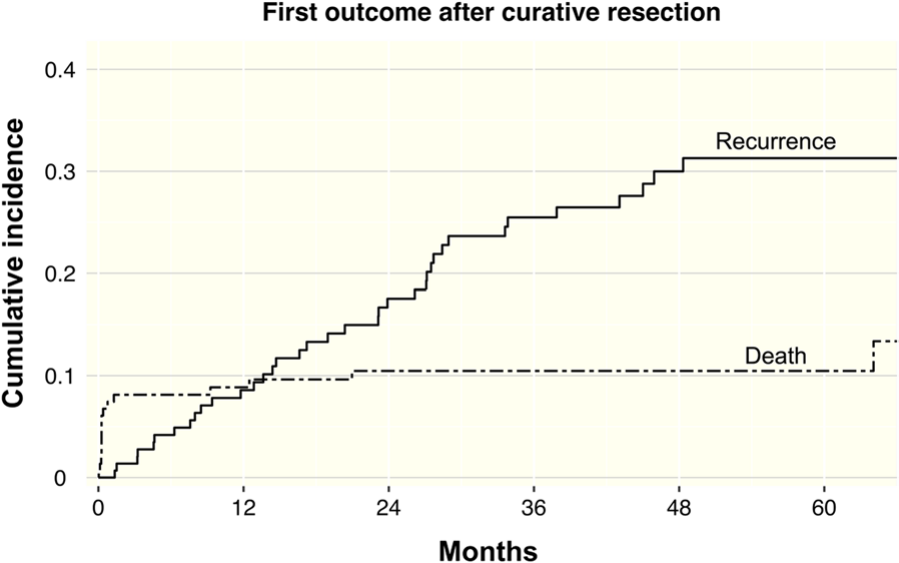
Cumulative incidence of first outcome after curative resection

Figure 2 provides a graphic display of CIF for recurrence and its competing risk of death. CIF for death rises quickly at first and then stabilizes, while recurrence shows a continuous, steadily rise. At 12, 24, 36 and 48 months after surgery CIF for death is 0.09, 0.1, 0.1, 0.1, compared to 0.09, 0.18, 0.25, 0.3 for recurrence.

### CIF by patient and disease characteristics

The CIF of recurrence by patient and disease characteristics is shown in Table 3. Estimates are given at 12, 24, 36 and 48 months following curative surgery. Highest recurrence at 48 months is seen at N-stage N2 (0.90), T-stage T4 (0.71), positive margins (0.69), vascular invasion (0.64), and adjuvant chemotherapy or radiation (0.60).

**Table 3:** Cumulative incidence of local/distant metastasis by potential risk factors for patients with colorectal cancer after resection surgery

Variable	Category	Cumulative incidence (months)			
		12	24	36	48
All patients		0.09	0.18	0.25	0.30
Age	< 60	0.11	0.23	0.31	0.37
	60–74	0.08	0.16	0.24	0.31
	≥ 75	0.08	0.16	0.24	0.24
Sex	Male	0.03	0.15	0.23	0.27
	Female	0.16	0.21	0.29	0.33
Tumour location	Colon	0.15	0.17	0.23	0.23
	Sigmoid/rectosigmoid	0.09	0.29	0.32	0.39
	Rectum	0.02	0.10	0.24	0.31
Tumor type	Adenocarcinoma	0.06	0.16	0.23	0.28
	Mucinous adenocarcinoma	0.32	0.32	0.48	0.48
Histological grade	Low/well differentiated	0.04	0.16	0.22	0.28
	Moderate	0.11	0.20	0.26	0.30
	High/poorly differentiated	0.07	0.07	0.29	0.29
T stage (depth)	T1/ T2	0.00	0.07	0.10	0.14
	T3	0.07	0.16	0.28	0.33
	T4	0.52	0.71	0.71	0.71
N stage (nodes)	N0	0.03	0.10	0.13	0.15
	N1	0.06	0.16	0.27	0.27
	N2	0.35	0.52	0.73	0.90
Vascular invasion	Absent	0.04	0.13	0.16	0.16
	Present/suspicious	0.22	0.28	0.51	0.64
Perineural invasion	Absent	0.07	0.14	0.22	0.25
	Present	0.13	0.28	0.37	0.44
Positive margins	Uninvolved	0.06	0.15	0.22	0.26
	One positive margin	0.36	0.43	0.58	0.69
Nodes examined	< 12	0.08	0.17	0.20	0.24
	≥ 12	0.09	0.18	0.27	0.31
Post-operative adjuvant therapy	None	0.08	0.14	0.14	0.16
	Chemotherapy or radiation	0.09	0.25	0.49	0.60

Table 4 shows results of testing for differences between pairs of CIFs using the Gray test. Statistically significant differences were found between N-stage N2 vs N0 (*p* < 0.001) and N1 (*p* < 0.001), among the 3 groupings of T-stage; T1/ T2 vs T3 (*p* = 0.038), T1/ T2 vs T4 (*p* < 0.001), T3 vs T4 (*p* < 0.001), between presence vs absence of vascular invasion (*p* < 0.001) and perineural invasion (*p* = 0.001), as well as adjuvant therapy vs no further treatment (*p* < 0.001).

**Table 4:** Gray test between pairs of cumulative incidence functions for recurrence

Variable	Groups compared	Gray test *p *value
Age (years)	< 60 vs 60–74	0.858
	< 60 vs ≥ 75	0.344
	60–74 vs ≥ 75	0.463
Sex	Male vs female	0.247
Tumour location	Colon vs sigmoid/rectosigmoid	0.199
	Colon vs rectum	0.796
	Sigmoid/rectosigmoid vs rectum	0.142
Tumor type	Adenocarcinoma vs mucinous	0.118
Histological grade	Low/well differentiated vs moderate	0.477
	Low/well differentiated vs high/poorly differentiated	0.933
	Moderate vs high/poorly differentiated	0.793
T stage (depth)	T1/ T2 vs T3	0.038
	T1/ T2 vs T4	< 0.001
	T3 vs T4	< 0.001
N stage (nodes)	N0 vs N1	0.119
	N0 vs N2	< 0.001
	N1 vs N2	< 0.001
Vascular invasion	Absent vs present/suspicious	< 0.001
Perineural invasion	Absent vs present	0.076
Positive margins	Uninvolved vs positive margin	0.001
Nodes examined	< 12 vs ≥ 12	0.852
Post-operative adjuvant therapy	None vs chemotherapy and/or radiation	< 0.001

### Modelling risk of recurrence

The Fine and Gray’s SD regression analysis was employed to model the hazard that corresponds to the CIF. The SHR for recurrence generated by the models are shown in Table 5, along with Wald test results indicating each covariate’s statistical significance. The SHR is the relative risk for a categorical covariate, defined as the ratio of subdistribution hazards for the actual group with respect to the baseline, with all other covariates being equal. If the covariate is continuous then the relative risk refers to the effect of a one unit increase in the covariate, with all other covariates being equal. In univariable modelling, SHR (95% CI) of T-stage T4 was 11.93 (3.74, 37.99), while T3 was 2.46 (1.04, 5.81) with reference T-stage T1/T2. The N-stage N2 SHR was 10.58 (5.17, 21.65) with reference to N0. Presence of vascular invasion was 4.27 (2.22, 8.21) relative to its absence, while SHR for positive margins was 3.63 (1.68, 7.84). The SHR for patients treated with adjunct chemotherapy or radiation was 3.85 (1.99, 7.46).

**Table 5:** Univariable subdistribution regression for recurrence under the competing risk of death

Variable	SHR^1^ (95% CI)	Wald test *p *value^2^
Age, per 10-yr increase from baseline 60 years	0.84 (0.63, 1.12)	0.23
Female	1.45 (0.77, 2.71)	0.249
Rectum (v. colon)	1.17 (0.52, 2.62)	0.138
Sigmoid/rectosigmoid (v. colon)	2.01(0.94, 5.56)	–
Mucinous (v. adenocarcinoma)	2.03 (0.81, 5.12)	0.132
Moderate grade (v. low grade)	1.31 (0.636, 2.7)	0.757
High grade (v. low grade)	1.12 (0.81, 5.12)	–
T3 stage v. T1/T2 stage	2.46 (1.04, 5.81)	< 0.001
T4 stage v. T1/T2 stage	11.93 (3.74, 37.99)	–
N1 stage v. N0 stage	2.02 (0.85, 4.8)	< 0.001
N2 stage v. N0 stage	10.58 (5.17, 21.65)	–
Vascular invasion	4.27 (2.22, 8.21)	< 0.001
Perineural invasion	1.82 (0.95, 3.49)	0.069
Positive margins	3.63 (1.68, 7.84)	0.001
Nodes examined (> 12)	1.07 (0.53, 2.17)	0.854
Post-operative chemotherapy or radiation	3.85 (1.99, 7.46)	< 0.001

^1^Subdistribution hazard ratio

^2^For the overall covariate

**Fig. 3:**
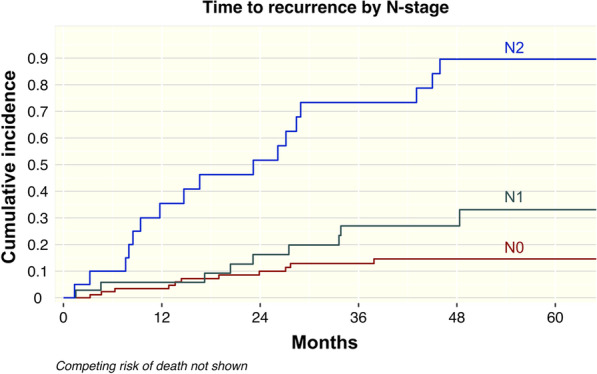
Cumulative incidence of time to recurrence by N-stage

The multivariable model was fit in three stages, using the forward selection method at each stage. All a-priori determined co-variates were included at the start. The data fit was assessed with AIC, BIC, and Schoenfeld residuals. Co-variables were dropped if they did not improve model fit until the model with the best fit with the least number of co-variates remained. Figure 3 shows the CIF for recurrence by each level of N stage.

## Discussion

A total of 44 patients in the study were lost to follow-up, which is one of the limitations of the study. Most patients were not followed after discharge due to advanced age, as 19 patients were ≥ 80 years old, with a mean age of 75.7 versus 69.2. A few of the others were lost to follow-up as they were repatriated back to their own geographic region. Disease characteristics of patients without follow-up showed no marked differences from patients under follow-up. Excluding these patients is not likely to introduce noteworthy bias to the study results. CRC local recurrence (n = 7), metastatic disease (n = 26), or both (n = 5) was found in a total of 38 patients (25.7%). The median time to recurrence was found to be 19.7 months and the median follow-up time in our study was 44.8 months, a time in which most recurrences occur. Sargent et al. demonstrated in a pooled analysis of patients with colon cancer that 80% of patients who experienced a recurrence were within the first 3 years [14].

The depth of tumor invasion along with the presence and degree of lymph node metastasis and vascular invasion have long been regarded as standard prognostic factors in CRC [15]. Some of these pathological features have defined our current widely accepted American Joint Committee on Cancer (AJCC) staging system for CRC. Our study examined the CIF of CRC recurrence, local or metastatic, by patient and disease characteristics. The factors showing highest CIF of recurrence at 48 months were N2 and T4 disease, positive margins, vascular invasion, and adjuvant chemotherapy or radiation. Statistically significant differences were found between N2 vs N0 and N1, and between T stage groupings: T1/T2 vs T3, T1/T2 vs T4, and T3 vs T4, as well as the presence of vascular invasion, perineural invasion, and adjuvant therapy. Numerous studies have demonstrated through multivariate analysis that N stage is a significant independent prognostic factor in CRC-related mortality [16]. T stage also has a clear impact on overall survival and found to be an independent prognostic factor in CRC [16–19]. Ueberrueck et al. found tumor invasion beyond the muscularis propria resulted in a substantial reduction in 10-year survival [16]. While the data in our study is not novel, it confirms these factors remain at the forefront of disease prognostication.

The results of this study present statistical data to corroborate what has become increasingly clinically evident within the field of colorectal oncology in recent years. Namely, that the traditional AJCC staging system, intended to help locate individual patients within a progressive outcome prediction scale, tends to inappropriately emphasize certain risk factors for recurrence over others. For instance, any N1 status results in an AJCC stage 3a or 3b designation, depending of T-stage, whereas a T4 status, in the absence of node positive and metastatic disease, earns a maximum designation of stage 2b. This would seem to indicate that N1 status is a stronger predictor of recurrence and poor outcome than T4 status. However, treating clinicians have increasingly recognized that outcomes for T4 colorectal adenocarcinoma patients (AJCC stage 2b) are markedly worse than for T1-2N1 patients (AJCC stage 3a), as these tumors recur more frequently, both locally or distantly. The present study poignantly highlights this fact by demonstrating that T4 tumors carry the highest hazard ratio (11.93) for recurrence of all the validated risk factors, including N stage, a fact that is not consistently identified in the literature. This emphasizes the importance of advanced T stage in clinical practice when considering the benefit of adjuvant therapy for risk reduction after curative intent surgery.

Our study found vascular invasion to be one of the factors with the highest CIF of recurrence. This is consistent with other studies that have shown vascular invasion to be an important prognostic factor in survival outcomes for CRC patients [18–21]. Courtney et al. found vascular invasion to be a prognostic factor independent of T or N stage, resulting in a decreased 5-year survival in CRC patients [22]. A study by Tsai et al. examined pathological features affecting disease recurrence and overall survival following curative resection in T2-4N0M0 CRC and found only T stage and vascular invasion were significant independent prognostic factors for relapse using Cox proportional hazards analysis [23]. There are mixed reports in regards to lymphatic invasion as a significant risk factor in subsequent disease recurrence and survival. The presence of lymphatic invasion has been correlated to poorer survival time [17] and a significant prognostic factor in CRC [20, 24], whereas other studies have not found as strong a prognostic association [21]. Numerous studies have found that lymphatic invasion correlates well with lymph node status, and therefore an important predictor of disease survival [18, 24, 25]. Perineural invasion has been found to be a significant prognostic factor in both univariate [23] and multivariate analyses with poorer survival rates in patients with the presence of perineural invasion [26].

While advancement in local and systemic therapy for CRC have been made in recent years since data acquisition for the present study (i.e. radiotherapy for rectal cancer, the addition of oxaliplatin for Stage III adjuvant therapy, immunotherapy for Stage IV disease, total neoadjuvant therapy, etc.), the recurrence risks for adenocarcinoma remain consistent, a fact this paper brings to light when compared with more contemporary data sets. The data from this cohort serves as a benchmark against which more modern results can be compared. Clearly, further research is needed to identify and refine understanding of risk factors which most significantly influence CRC patient outcomes (i.e. ctDNA).

## Data Availability

The datasets analysed during the current study are available from the corresponding author on reasonable request.

## Abbreviations

CRC: Colorectal cancer
CIF: Cumulative incidence function
SD: Subdistribution
IQR: Intra-quartile range
SHR: Subdistribution hazard ratio
AIC: Akaike information criterion
BIC: Bayesian information criterion
AJCC: American Joint Committee on Cancer
